# Nonmetallization and band inversion in beryllium dicarbide at high pressure

**DOI:** 10.1038/srep26398

**Published:** 2016-05-20

**Authors:** Henan Du, Wanxiang Feng, Fei Li, Dashuai Wang, Dan Zhou, Yanhui Liu

**Affiliations:** 1 Department of Physics, College of Science, Yanbian University, Yanji 133002, China; 2 School of Physics, Beijing Institute of Technology, Beijing 100081, China; 3 Laboratory of Clean Energy Technology, Changchun University of Science and Technology, Changchun 130022, China; 4 Beijing Computational Science Research Center, Beijing 10000, China

## Abstract

Carbides have attracted much attention owing to their interesting physical and chemical properties. Here, we systematically investigated global energetically stable structures of BeC_2_ in the pressure range of 0–100 GPa using a first-principles structural search. A transition from the ambient-pressure α-phase to the high-pressure β-phase was theoretically predicted. Chemical bonding analysis revealed that the predicted phase transition is associated with the transformation from *sp*^2^ to *sp*^*3*^ C-C hybridization. The electrical conductivity of the high-pressure phase changed from a metal (α-phase) to a narrow bandgap semiconductor (β-phase), and the β-phase had an inverted band structure with positive pressure dependence. Interestingly, the β-phase was a topological insulator with the metallic surface states protected by the time-reversal symmetry of the crystal. The results indicate that pressure modulates the electronic band structure of BeC_2_, which is an important finding for fundamental physics and for a wide range of potential applications in electronic devices.

Carbides draw much attention because of their unusual physical and chemical properties^1^,^2^,^3^. The transition between various carbides has long been a topic of interest because these phases have very different electronic properties, owing to the change from *sp*^2^ to *sp*^3^ carbon hybridization during the transition from graphite to diamond under sufficient pressure. Alkaline earth metal carbides have been the focus of research for more than half a century, and have been the subject of extensive theoretical and experimental studies of advanced functional materials^4^,^5^,^6^. Many alkaline earth metal dicarbides possess a linear crystal structure at normal condition. Previous experimental results show that magnesium dicarbide (MgC_2_) crystallizes in a tetragonal lattice with the space group *P*4_2_/*mnm*^7^,^8^. Experimental and theoretical studies reported that the structure of CaC_2_ belongs to the *I*4/*mmm* space group. Recently, the structural evolution of CaC_2_ was determined using first principles calculations^9^. Especially, the high-pressure phase of CaC_2_ (*Immm*) of the intercalated graphite structure has a high superconducting critical temperature (Tc) of 7.9–9.8 K, which is comparable to Tc CaC_6_ (11.5 K)^10^,^11^,^12^,^13^,^14^,^15^. However, there are few studies exploring the crystal structure of BeC_2_, and its high-pressure structures are poorly understood, which will impede further exploration of this important class of material. A pressing task is to understand the crystal and electronic structures of this special matter under external pressure condition that may alter the underlying fundamental physics, which has motivated us to carry out the work.

In this study, we therefore explored the hitherto unknown high-pressure phase diagram of beryllium dicarbide via a first-principles swarm-intelligence structure search and established a comprehensive understanding of the high-pressure evolution of BeC_2_. Our work shows that BeC_2_ crystallizes in the monoclinic *C*2/*m* (α-phase) structure at ambient pressure and undergoes isostructural transitions to the β-phase at high pressure. We identified two thermodynamically stable phases associated with the transition from *sp*^2^ to *sp*^3^ C-C hybridization and predicted that the β-phase is a topological insulator. Our result represents a significant step toward understanding the structural and electrical behavior of alkaline earth metal carbides under extreme condition.

## Result and Discussion

### Crystal structures of BeC_2_

**Figure 1:**
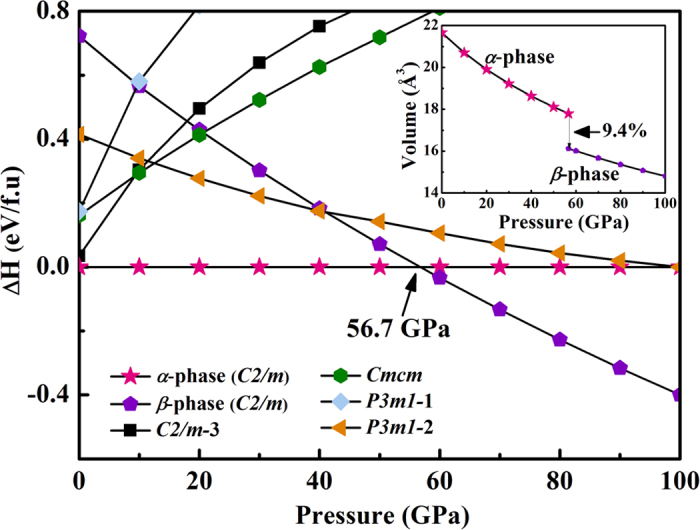
Enthalpies per formula unit of various structures as a function of pressure with respect to the ambient pressure of the α-phase. Inset: Calculated equations of states for the predicted structures.

We performed structure prediction for BeC_2_ with variable-cell simulation cell sizes of 1–4 formula units at pressure of 0–100 GPa. At ambient pressure, our simulation reveals that the *C*2/*m* phase denotes as the *α*-structure/phase has lower enthalpies than all the other candidates, indicating that the *α*-structure is in the thermodynamic ground state. Over the whole pressure range, a new low-enthalpy structure denotes as the β-structure/phase is explored which has the same space group as the *α*-structure. The enthalpy-pressure relationship for the candidate structures is shown in Fig. 1. It has been seen that, for compressed BeC_2_, the ambient-pressure *α*-phase is the most stable structure below 56.7 GPa, but the β*-*phase becomes more favorable from 56.7 to 100 GPa. The pressure evolution of the unite cell volume of BeC_2_ in the α- and β-structure is depicted in inset of Fig. 1. The volume collapses about 9.4% around 56.7 GPa, indicating the first-order nature of the phase transition in BeC_2_. No imaginary frequency is observed throughout the whole Brillouin zone, which indicates the two novel phases are dynamically stable in the pressure region from this study.

**Figure 2:**
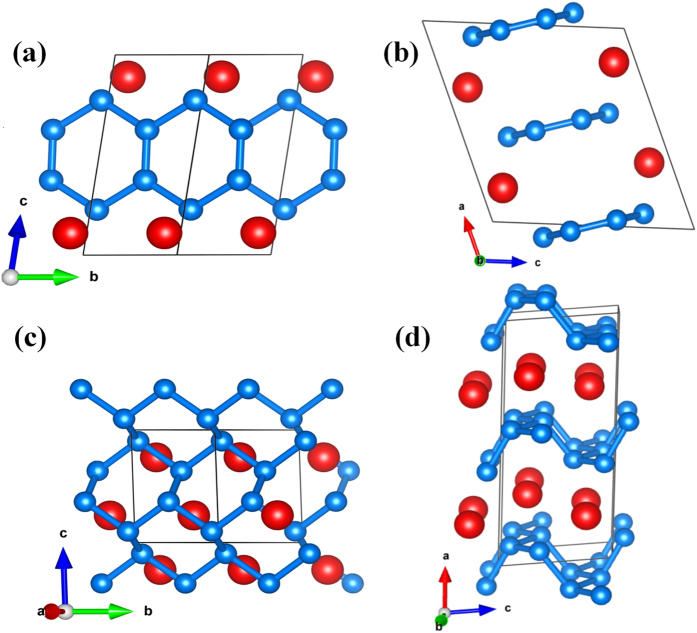
Structures of predicted stable BeC_2_ crystal. (**a**,**b**) α-phase and (**c**,**d**) β-phase. The large (red) and small (blue) spheres are Be and C atoms, respectively.

The atomic arrangements of the competing structures are shown in Fig. 2. The calculated lattice constants of the ambient-pressure are calculated. The α*-*phase has a monoclinic symmetry, with *a* = 6.223 Å, *b* = 2.535 Å and *c* = 5.904 Å, β = 111.5° at ambient pressure. The Be and C atoms occupy Wyckoff 4*i* position in the unit cell with Be at (0.672, 0.5, 0.11), C at (0.431, 0.5, 0.227) and (0.467, 1.000, 0.374). The α-phase is similar to that the recently predicted for MgC_2_ of *C*2*/m* phase with carbon atoms polymerized into ribbon with a six-membered ring. Planes of Be atoms separate the hexagonal honeycomb layers of carbon atoms, which have the nearest distance with the outer C atom of the ribbon.

**Figure 3:**
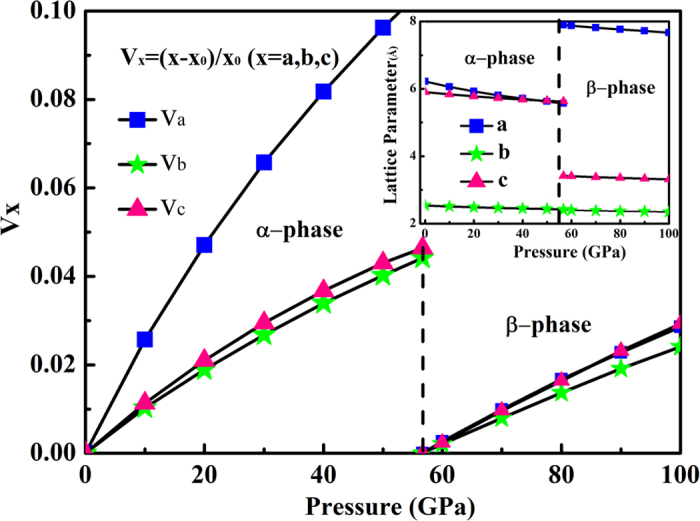
Calculated lattice parameter change on the pressure in the α- and β- phase. (X_0_ indicate the lattice parameters for the α- and β- phase at 0 GPa and 56.7 GPa, respectively). Inset: The Lattice parameter in the α- and β-phase as a function of pressure.

The β-phase has a monoclinic symmetry, with lattice parameters of *a* = 7.879 Å , *b* = 2.404 Å, *c* = 3.415 Å, and β = 84.12° at 56.7 GPa. The Be and C atoms occupy Wyckoff 4*i* positions in the unit cell with Be at (0.212, 0.5, 0.758), C at (0.420, 0.5, 0.9) and (0.418, 1.000, 0.637). It is obvious that the β-phase consists of bulky honeycomb-layer of carbon atoms separated by planes of Be atoms. Figure 3 shows the calculated lattice parameter versus pressure curves for the α- and β-phase. In α-phase, the slope of *a* is much more steeper than that of *b* and *c*. The results show that the inter-atomic forces among the interlayer molecules are weaker than the forces among in the intralayer molecules. For the β-phase, the slopes in different axes are nearly identical. In the inset of Fig. 3, the change of β-phase induced by the pressure is attributable to the elongating of the interlayer (*a* axis) and shortening of the intralayer (*c* axis) in α-phase, which also caused the well-organized six-ring carbon rings collapse into the puckered quasi 2-dimension graphite sheet. Due to the occurrence of graphite sheet between neighboring Be atomic layers, the β-phase can be regarded as one of the puckered graphite intercalation compounds.

### Nonmetallization of BeC_2_

**Figure 4:**
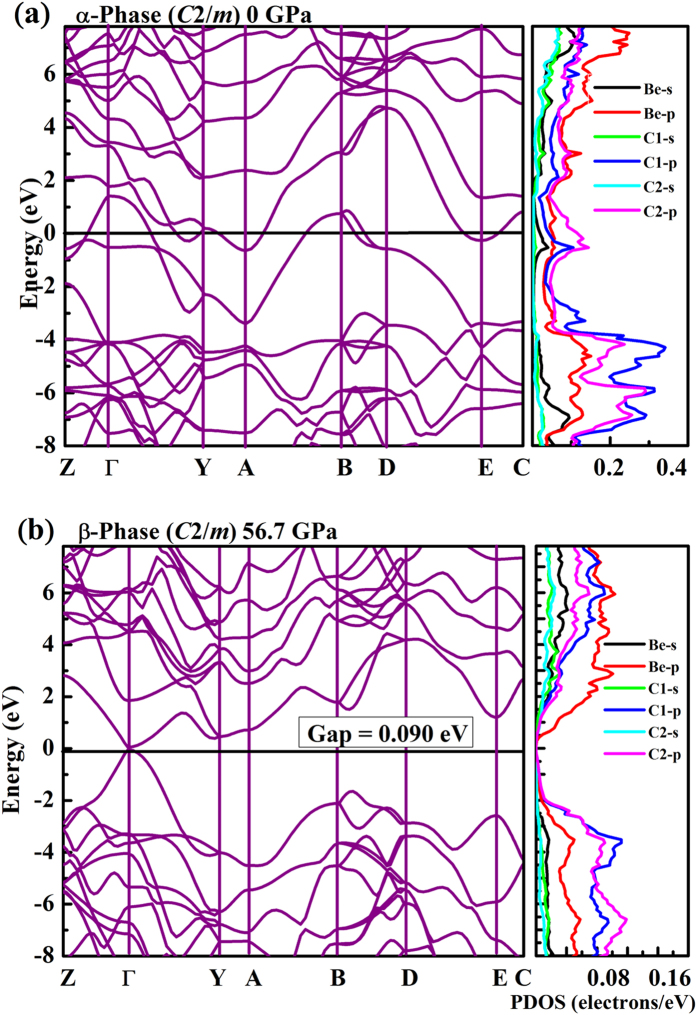
Electronic band structure and projected DOS of BeC_2_. (**a**) α-phase and (**b**) β-phase. Zero of energy is at the Fermi level.

The calculated band structure and projected density of states (DOS) are shown in Fig. 4. The predicted overlap between the conduction and the valence bands shows the α-phase with *sp*^2^ hybridization is metallic, along with Be- and *C- p* electrons near the Fermi level. At 56.7 GPa, the β-phase characterized by *sp*^3^ hybridization becomes an insulator with a direct band gap of 0.03 eV (without spin-orbit coupling). Generally, metals under pressure exhibit decreasing interatomic distance and increasing metallic behavior, whereas BeC_2_ undergoes an unexpected and often counterintuitive pressure-induced metal-to-insulator transition. This surprising behavior can also be seen in Sodium which shows an optically transparent phase at 200 GPa^16^.

**Figure 5:**
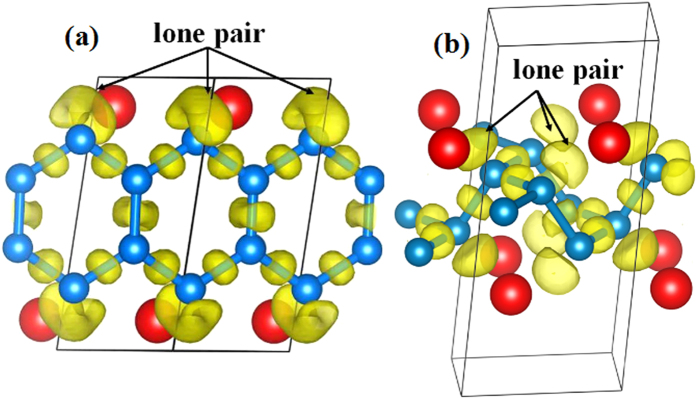
ELF isosurface (value = 0.8) illustrated on a plane containing C_6_ rings in BeC_2._ (**a**) α-phase and (**b**) β-phase.

To examine the nature of these bonding states, we plotted the electron localization function (ELF) isosurfaces of BeC_2_ (Fig. 5)^17^. The α-phase contains a ribbon of six-member carbon rings, which have *sp*^2^-like properties. Each inner C atom forms three C-C covalent bonds and each outer C atom has one electron lone pair along with two C-C covalent bonds. The remaining 2*p*_*z*_ electrons of the C_6_ ring form a delocalized *π* system. In the β-phase, the puckered honeycomb-layered structure lacks a system of delocalized *π* bonds with mobile electrons and has more localized electrons resulting in *sp*^3^ hybridization. Each C atom has one electron lone pair and three covalent C-C bonds, resembling diamond structure, accompanied by the appearance of an insulator state. The significant evolution of electronic structures from the *sp*^2^ to *sp*^3^ hybridization of the carbon atoms between the α- and β-phases is similar to the transformation of graphite to diamond under sufficient compression.

### Band inversion and topological insulator state in β-phase

**Figure 6:**
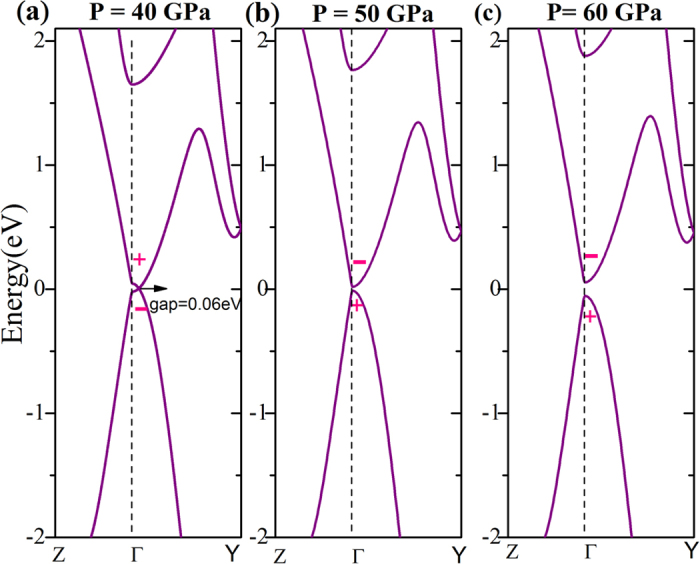
Electronic band structures for the β-phase under different pressures. The topological phase in (**a**) is a topological insulator, those in (**b**,**c**) are normal insulators. Zero of energy is at the Fermi level.

Remarkably, the size of the band gap of the β-phase depends positively on pressure in Fig. 6. This peculiar behavior stems from the requirement for symmetry thus forbidding the crossing inverted conduction minimum and valence maximum at Γ point; consequently, the conduction minimum and valence maximum will repel each other more strongly under higher pressure. It is important to check whether the β-phase is a topological insulator at high pressure. Due to the presence of inversion symmetry, the parity criterion can be used to justify the topological properties of the β-phase^18^,^19^,^20^. Following the method addressed in ref. 18, we calculated the parities *δ*_1_ at eight time-reversal invariant momenta (TRIMS) (*i* = 1–8) in three-dimensional Brillouin zone in Table 1. At 40 GPa, only the Γ point has negative parity, whereas the other TRIMS have positive parity. At 50 and 60 GPa, all TRIMS have positive parity. This can also be observed from the band structures of the β-phase around the critical pressure of the phase transition, as shown in Fig. 6. One can clearly observe that the parities of band edges are exchanged when above 40 GPa. The Z_2_ topological invariants can then be evaluated from the parities using the following equations,


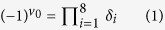



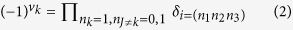


The Z_2_ numbers are 1; (111) for 40 GPa and 0; (000) for 50 and 60 GPa. Together with the evolution of band energy as a function of pressure (Fig. 6), we can confirm that the β-phase is a strong topological insulator when below 40 GPa, whereas it is a normal insulator when above 50 GPa.

## Conclusion

In summary, we have determined the high-pressure structural evolution of BeC_2_ by using the particle swarm optimization technique with first-principles electronic structure calculation. We predicted a pressure-induced phase transition from the α- to β-phase at 56.7 GPa. The structural, dynamical, and electronic properties of BeC_2_ under pressure were systemically investigated. The electrical calculation shows that the β-phase has an inverted band structure with a positive pressure dependence. Interestingly, the β-phase is a topological insulator with metallic surface states protected by the time-reversal symmetry of the crystal. These results show that pressure has a strong effect on the fundamental crystal and electronic structure of BeC_2_, and that pressure tuning of the electronic properties offers an effective tool to modulate a wide range of physical properties for its potential applications.

## Method

We performed structure predictions through a global minimization of free energy surfaces merging *ab initio* total-energy calculations implemented in CALYPSO (crystal structure analysis by particle swarm optimization) code^21^,^22^,^23^,^24^,^25^. The method has successfully predicted the high-pressure structures of various systems, ranging from elements to binary and ternary compounds^26^,^27^. The first-principles energetic calculations were carried out using density functional theory (DFT) with the Perdew-Burke-Ernzerhof of exchange-correlation as implemented in the Vienna *ab initio* simulation package (VASP)^28^. The projector augmented wave method has been adopted, with 2*s*^2^ and 2*s*^2^ 2*p*^2^ treated as valence electrons for Be and C atoms, respectively^29^,^30^. For Brillouin zone integration, we used the Monkhorst-Pack scheme and checked convergence of ground state calculations with uniformly increasing *k*-point meshes for each structure^31^. We used cutoff energy of 800 eV for the expansion of the wave function into the plane-wave basis-set. Monkhorst-Pack *k*-point meshes with a grid of 0.03 Å^−1^ for Brillouin zone sampling were chosen to achieve the total energy convergence of less than 1 meV/atom. The phonon calculations were carried out by using a supercell approach as implemented in the PHONOPY code^32^. This method uses the Hellman-Feynman forces calculated from the optimized supercell through VASP. Ultrasoft pseudopotentials for C and Be were used, and convergence tests concluded that suitable calculation parameters are 80 Ry for kinetic energy cutoff, 20 × 20 × 12 *k*-point sampling mesh, and 5 × 5 × 3 *q*-mesh in Brillouin zone.

## Additional Information

**How to cite this article**: Du, H. *et al*. Nonmetallization and band inversion in beryllium dicarbide at high pressure. *Sci. Rep.* **6**, 26398; doi: 10.1038/srep26398 (2016).

## Figures and Tables

**Table 1:** Parities at eight time-reversal invariant momenta and Z_2_ topological invariants for tne β-phase around the critical pressure of the phase transition.

**Pressure** (**GPa**)	**Parities**								**Z**_**2**_ **index**
	(**0, 0, 0**)	(**0, π, 0**)	(**π, 0, 0**)	(**π, π, 0**)	(**0, 0, π**)	(**0, π, π**)	(**π, 0, π**)	(**π, π, π**)	
40	−	+	+	+	+	+	+	+	1; (111)
50	+	+	+	+	+	+	+	+	0; (000)
60	+	+	+	+	+	+	+	+	0; (000)
